# Development Of Multiplexed Electroanalytical Biosensing Platforms For The Clinical Diagnosis Of Alzheimer’s Disease

**DOI:** 10.1002/alz.086184

**Published:** 2025-01-09

**Authors:** Ana Montero‐Calle, Maria Garranzo‐Asensio, Alejandro Valverde, Pablo San Segundo‐Acosta, Eloy Povedano, Rebeca M. Rodríguez Torrente, Mónica Vázquez, Vicente Mas, Susana Campuzano, Rodrigo Barderas

**Affiliations:** ^1^ Instituto de Salud Carlos III, Madrid, Madrid Spain; ^2^ Universidad Complutense de Madrid, Madrid, Madrid Spain

## Abstract

**Background:**

Alzheimer’s disease (AD) is the most common neurodegenerative disease worldwide and the leading cause of dementia in the elderly. New approaches to study AD are still needed to identify and validate blood‐based diagnostic biomarkers that could be useful for its early diagnosis. Circulating autoantibodies (AAbs) and their target proteins (autoantigens) are promising candidate biomarkers to aid in AD early diagnosis. Thus, our objective was to validate here the potential of electrochemical biosensors for the diagnosis of AD based on the measurement of plasma autoantibodies previously described as specific of AD.

**Methods:**

An immunoplatform based on the use of Halo‐MBs (magnetic beads) modified with validated AD autoantigens (previously identified by phage microarrays, immunoprecipitation, or planar protein‐epitope signature tag (PrEST) arrays, or as aberrant AD proteins) was developed. AD autoantigens were cloned and expressed in mammalian cells as HaloTag fusion peptides or proteins. The oriented covalent immobilization of the HaloTag peptides or proteins to the surface of the MBs allowed for the efficient and selective capture of the corresponding AAbs from plasma samples. Then, signal was developed by amperometric transduction on disposable electrodes using the H_2_O_2_/HQ (hydroquinone) system after enzymatic labelling of the captured human IgGs with an HRP‐conjugated secondary antibody.

**Results:**

With the developed methodology, plasma autoantibodies against two aberrant AD‐associated peptides, four peptide autoantigens identified by Phage Microarrays, and three full‐length AD autoantigens identified by multiomics analyses were used to construct the first multiplexed bioplatform described to date, based on the use of this type of receptors expressed in mammalian cells, to evaluate its potential for AD diagnosis by targeting their associated autoantibodies. After optimization of key variables in the development of the multiplexed biosensing bioplatform, its analytical operational characteristics demonstrated a highly significant clinical diagnostic potential in a single test.

**Conclusions:**

Our results suggest the possibility of reliably and minimally invasive diagnose AD by using amperometric biosensing platforms detecting AD plasma autoantibodies against specific autoantigens with a high sensitivity and specificity. Additionally, these multiplexed bioplatforms could be used as point‐of‐care (POC) devices for the clinical diagnosis of AD by liquid biopsy and in less than 90 minutes.

